# Discovery and analytical assessment of urinary miRNA biomarkers for cervical cancer using advanced small RNA sequencing

**DOI:** 10.1016/j.tranon.2026.102857

**Published:** 2026-06-16

**Authors:** Mengfei Xu, Mariano A. Molina, Cristina Gómez-Martín, Maaike M.H.S. Dekker, Saba Ghasemi, Johan de Rooij, D. Michiel Pegtel, Nienke E. van Trommel, Renske D.M. Steenbergen

**Affiliations:** a Amsterdam UMC, location Vrije Universiteit Amsterdam, Department of Pathology, De Boelelaan 1117, Amsterdam, Netherlands; b Cancer Center Amsterdam, Imaging and Biomarkers, Amsterdam, Netherlands; c You2Yourself B.V., Enschede, Netherlands; d Antoni van Leeuwenhoek/Netherlands Cancer Institute, Department of Gynecologic Oncology, Center of Gynecologic Oncology Amsterdam, Amsterdam, Netherlands

**Keywords:** miRNA, Urine, Non-invasive, Liquid biopsy, Small RNA sequencing, Cervical cancer

## Abstract

Cervical cancer is primarily caused by persistent infection with high-risk human papillomavirus (hrHPV), which alters host RNA expression, including regulatory microRNAs (miRNAs). Because urine is emerging as a convenient biosource for cancer detection, we evaluated the feasibility of urinary miRNAs as non-invasive biomarkers for cervical cancer. We optimized miRNA isolation from whole urine and urine fractions using commercial RNA extraction kits, spike-in controls, and digital nanoplate PCR. Following identification of optimal conditions, IsoSeek small RNA sequencing was performed to profile miRNA expression in urine from women with cervical cancer (*n* = 20) and age-matched controls (*n* = 20). Differentially expressed candidates were then analytically confirmed by RT-qPCR. The Norgen RNA isolation kit applied to whole urine provided the most robust miRNA recovery. Sequencing identified significantly altered urinary levels of hsa-miR-143–3p and hsa-miR-204–3p in cervical cancer. The ratio of these miRNAs showed strong discriminatory performance for detecting cervical cancer using IsoSeek (AUC = 0.93) and remained robust after RT-qPCR analysis in the same cohort (AUC = 0.84). These findings support the feasibility of using urinary miRNA profiling for non-invasive biomarker discovery and highlight the hsa-miR-143–3p/hsa-miR-204–3p ratio as a promising candidate marker for cervical cancer detection.

## Highlights

• Optimized workflow enables sensitive miRNA detection from whole urine.
• IsoSeek enables high-sensitivity profiling of urinary miRNAs.
• Urinary miR-143–3p and miR-204–3p are dysregulated in cervical cancer.
• miR-143–3p/miR-204–3p ratio detects cervical cancer with promising discriminatory performance.

## Graphical abstract

**Figure:**
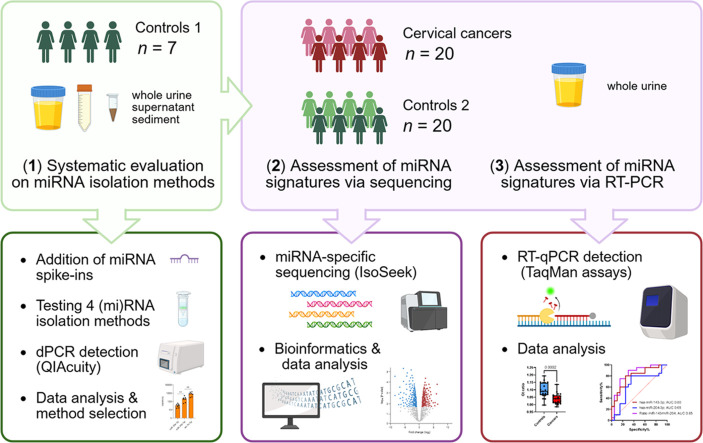

## Introduction

Cervical cancer is the fourth leading cause of cancer-related deaths among women worldwide. In 2022, over 660,000 women were diagnosed, and 345,000 died of cervical cancer, with the majority of deaths occurring in low- and middle-income countries [1]. The incidence of cervical cancer has been reduced by the broad application of a population-based screening, aiming at the early detection of cervical cancer and precancer. Current screening strategies mostly involve primary high-risk human papillomavirus (hrHPV) testing followed by cytology triage of hrHPV-positive women either or not combined with HPV genotyping. However, with vaccinated women entering the screening program and the growing use of cervicovaginal self-sampling, which precludes cytology triage, current triage strategies are suboptimal [2]. This highlights the need for alternative triage strategies for HPV-positive women.

Beyond advancements in screening methods, participation rate is another crucial factor affecting the effectiveness of cervical cancer screening. Minimally invasive samples, such as self-collected cervicovaginal swabs and urine, are considered patient-friendly and easy to collect at home-based settings, with urine samples being particularly preferred by women [3]. While multiple studies have demonstrated the value of HPV testing in urine, research on triage testing of urine remains limited. The development of objective triage tests for HPV-positive self-collected samples represents an important unmet clinical need, particularly with the increasing use of cervicovaginal self-sampling approaches in cervical screening programs and the growing interest in urine sampling. Our recent studies have shown that DNA methylation assays applied to urine samples exhibit good sensitivity for cervical (pre)cancer detection [4,5], though room for improvement remains.

MicroRNAs (miRNAs) are small non-coding RNAs that regulate gene expression at the post-transcriptional level. Increasing evidence has demonstrated miRNA dysregulation during HPV infection and cervical carcinogenesis, revealing its potential as biomarkers for cervical (pre)cancer diagnosis and risk stratification [[6], [7], [8]]. Due to their stability in bodily fluids, we hypothesize that miRNAs may act as non-invasive biomarkers for cervical cancer detection in urine. Previous studies have explored the potential of miRNAs as biomarkers for the detection of various urological cancers in urine [9], but research on cervical cancer detection is limited [10]. Isolating and profiling miRNAs from liquid biopsies, such as urine, represents a challenge for biomarker research. Several studies have tested various miRNA isolation methods using diverse urine starting materials, such as urine supernatant [11], urine extracellular vesicles [12], or filtered urine [13]. However, there is a lack of consensus on the optimal miRNA isolation method for all urine fractions (whole urine, supernatant, and sediment) as well as on the best approach for miRNA detection and quantification. This gap underscores the need for comparative studies to establish standardized protocols.

This study aims to evaluate the feasibility of detecting cervical cancer using urinary miRNAs as biomarkers. We compare four different miRNA isolation methods and assess miRNAs using sequencing- and PCR-based techniques. Next to it, we employ IsoSeek, a sequencing protocol established specifically for small RNA sequencing and optimized for advanced detection of miRNAs in low-input samples such as urine [14]. Our research identifies the most effective miRNA isolation method for urine collected at home-based settings and demonstrates the potential of urinary miRNAs as biomarkers for cervical cancer detection.

## Material and methods

### Study population

In this study, a total of 52 urine samples were collected from healthy women (*n* = 32) and patients diagnosed with cervical cancer (*n* = 20). All participants were instructed to collect full void urine, regardless of collection time and personal hygiene. Two series of urine samples from healthy controls were collected within the Urine Controls (URIC) biobank without knowledge of HPV status. Control series 1 (*n* = 7) was only used for technical optimization, control series 2 (*n* = 25) was used as age-matched controls for cancer samples. Urine samples from women diagnosed with cervical cancer (*n* = 20) were collected within the SOLUTION 1 study after confirmation of cancer diagnosis by histopathology and before treatment [4]. All participants provided written informed consent. Ethical approval was provided by the Medical Ethical Committee of the VU University Medical Center (URIC biobank, reference number: 2018.657; and SOLUTION studies, reference number 2016.213; Trial registration ID: NL56664.029.16).

### Urine sample collection and processing

Urine samples from the URIC biobank and SOLUTION 1 study were collected at home in three 30 mL tubes containing 2 mL of 0.6 M ethylenediaminetetraacetic acid (EDTA) and sent to the laboratory by regular mail, as established previously [15]. Upon arrival, the samples were aliquoted in 15 mL tubes and centrifuged at 3000 g × 10 min to obtain the supernatant and sediment fractions. All three fractions: whole urine, supernatant, and sediment were stored at −20 °C.

### RNA isolation, quantity, and quality

For performance comparison, four different miRNA isolation methods were used according to manufacturer’s instructions: the miRNeasy mini kit (abbreviated to miRNeasy_m in this study; Cat#217,084, Qiagen, Hilden, Germany), the miRNeasy Serum/Plasma Advanced Kit (miRNeasy_sp; Cat#217,204, Qiagen), the Urine Total RNA Purification Maxi Kit Dx (Slurry Format) (Norgen; Cat#Dx29600, Norgen Biotek Corp, Ontario, Canada) and the TRIzol™ Reagent (TRIzol; Cat# 15,596,026, Thermofisher Scientific, Waltham, MA, USA). To enable assessment of isolation efficiency, all samples were premixed with 30 µL of 6 × 10^6^ copies/µL cel-miR-76–3p mimic (*mir*Vana®miRNA mimic, MC10594, ThermoFisher Scientific) as an isolation spike-in prior to RNA extraction. Following urine processing, different input volumes were used: 5 mL of whole urine, 5 mL of urine supernatant, or urine sediment (15 mL original volume) were isolated with Norgen; and 1 mL of whole urine, 1 mL of urine supernatant, or urine sediment for miRNeasy_m, miRNeasy_sp, and TRIzol isolation. For all RNA isolation methods, 100 µL of 3 M Sodium Acetate (pH 5.2) was added as an RNA carrier for RNA precipitation. Concentration and purity of total RNA were measured by Nanodrop 1000 (Thermo Fisher Scientific). miRNA-specific quantification was performed using Qubit™ microRNA Assay Kits and Qubit 4 Fluorometer (Thermo Fisher Scientific). For small RNA sequencing and RT-qPCR, whole urine samples from the URIC biobank and the SOLUTION-1 study were processed using the Norgen Total RNA Purification Kit.

### Small RNA sequencing (IsoSeek)

Small RNA libraries and sequencing were made with an optimized protocol, based on the IsoSeek workflow [14,16]. In brief, small RNA libraries were prepared using the NEBNext® Small RNA Library Prep Set for Illumina® (New England Biolabs, Ipswich, MA, USA) with custom-designed 5′-5 N and 3′-5N-adaptors. Libraries were prepared from 4 µL RNA as input, adding 1:50 diluted adaptors and RT-primers (3′-adaptor 100 nM and 5′-adaptor 225 nM), spike-in sets (*n* = 26, 100 pM of each). 3′-adaptor ligation was performed prior to RT-primer hybridization, followed by 5′-adaptor ligation and reverse transcription. Libraries were amplified and purified using Monarch PCR & DNA Cleanup Kit (New England Biolabs), then eluted in 31 µL H_2_O. Gel size selection was performed with an automated BluePippin system (Sage Science Inc., Beverly, MA, USA, 3% Agarose gel cassette) following the manufacturer's instructions. In short, libraries were loaded onto a pre-cast gel cassette with an internal marker. They were then run on the BluePippin with an elution range of 127 – 155 bp. Libraries were then quantified using a QC-PCR, with the protocol adjusted according to the KAPA Library Quantification Kit (Roche, Basel, Switzerland). PCR was performed in 10 µL reactions containing 2 µL diluted (1,000x and 10,000x) libraries, 6 µL Primer Premix/KAPA SG Fast, and 2 µL H_2_O. Reactions were conducted using the LightCycler 480 System (Roche). Libraries were then pooled equimolar and underwent sequencing on an Illumina NextSeq2000 run (PE 50 bp, ∼10 M raw reads per sample) per standard protocols by the Utrecht sequencing facility (USEQ, Utrecht, The Netherlands).

### IsoSeek data processing and miRNA profiling

Data processing and analysis of small RNA sequencing data obtained from IsoSeek were performed using R v4.3.2. For pre-processing, adapter-trimmed read mapping was performed using the latest version of the sRNAbench command line tool [17]. Default parameters were used for all analysis steps and miRBase (v22) [18] was used as a miRNA reference. Quality control was carried out using mirnaQC [19] to remove technical differences. No samples were identified as major outliers during quality assessment, and all 40 samples were retained for downstream analyses. Row reads, miRNA-mapped reads are shown in **Supplementary Table 1.**

Raw counts for captured miRNAs generated by sRNAbench were used. All downstream processing and statistical analyses were conducted in R v4.3.2. Normalization and differential expression analysis were performed using DESeq2 v1.42.1 [20]. miRNAs with ≥10 counts in at least two of the 40 samples were analyzed. Counts were normalized by estimating size factors with the median-of-ratios method to correct for differences in sequencing depth and RNA composition between samples. This normalization strategy was selected because it is robust to sequencing depth variation and compositional differences commonly observed in small RNA sequencing datasets. Differential expression analysis was performed by modeling count data using a negative binomial distribution and evaluating statistical significance with Wald tests. P-values were adjusted for multiple testing using the Benjamini–Hochberg procedure. miRNAs with an adjusted p-value (false discovery rate, FDR) ≤ 0.1 were considered differentially expressed and were further classified as upregulated or downregulated based on an absolute log2 fold-change threshold of 1.

### Reverse transcription – quantitative polymerase chain reaction (RT-qPCR)

Reverse transcription was performed using the Taqman microRNA Reverse Transcription Kit (Thermo Fisher Scientific). The manufacturer’s instruction was adapted to the use of our lab for multiplex reverse transcription [7]. Twenty ng of miRNA was reverse transcribed in 17 µL reactions containing 0.5 µL of each miRNA primers, 1 µL cel-miR-54–3p mimic (6 × 10^6^ copies/uL, *mir*Vana®miRNA mimic, MC10279, ThermoFisher Scientific) as RT-spike-in, 0.5 µL 10x RT buffer, 0.1 µL dNTP, 0.06 µL RNase inhibitor (20 U/µL), 1 µL MultiScribe Reverse Transcriptase, and 1.34 µL RNase free water. The same RNA isolates for sequencing were also utilized for RT-qPCR, with the exception of five URIC controls that had insufficient input material; these were replaced by five alternative isolates from the URIC cohort. The sample input amount used in the reaction was available for all twenty cancer samples. All individual miRNAs were measured using TaqMan™ MicroRNA Assay (Catalog: 4427,975, Thermo Fisher Scientific), miRNA names and Assay ID are listed in **Supplementary Table 2.** Quantitative PCR (qPCR) was performed in compliance with the minimum information for publication of quantitative PCR experiments (MIQE) guidelines [21]. Reactions were performed in a 10 µL volume containing 5 µL 2x Taqman Universal Mastermix II no UNG, 0.5 µL miRNA-specific Taqman assays, 3.5 µL H2O, and 1 µL cDNA. qPCR reactions were conducted and analyzed using the ViiA7 Real-Time PCR System (Thermo Fisher Scientific).

### Digital-nanoplate PCR (dPCR)

dPCR was performed according to the minimum information for publication of digital PCR experiments (dMIQE) guidelines [22]. Reactions were performed in a 12 µL volume containing 3 µL 4x Probe PCR Master Mix (Qiagen), 1.2 µL miRNA-specific Taqman assays (Thermo Fisher Scientific), 5.8 µL H2O, and 2 µL cDNA. dPCR reactions were conducted in QIAcuity Nanoplate (8.5 K 24-well or 96-well) using QIAcuity One 5plex and analyzed using QIAcuity Software Suite 2.5.0.1 (Qiagen). The fluorescence intensity (RFU) threshold of dPCR was set to eliminate negative partitions. For most miRNAs, a threshold of 60 RFU was set.

### Statistical analysis

RNA and miRNA yield and spike-in levels across samples and urine fractions were compared using a Friedman test [23]. In case of a significant Friedman test (*p* < 0.05), a Benjamini-Hochberg multiple testing correction [24] was performed between each group, and q-values (adjusted p-values) were calculated and displayed in figures. Expression levels of endogenous miRNAs were shown as copies/µL reaction and presented in box plots. Comparisons across differentially abundant endogenous miRNAs were performed using a Kruskal-Wallis test, followed by a Benjamini-Hochberg multiple test correction when significant (*p* < 0.05). Significant differences indicated by the adjusted q-values (adjusted p-values) between groups are denoted by * *q* < 0.1, ** *q* < 0.05, *** *q* < 0.01, **** *q* < 0.001. Given the exploratory and discovery-oriented nature of this study and the low-input characteristics of urinary miRNA sequencing, an FDR threshold of 0.10 was selected to improve sensitivity for candidate biomarker identification while controlling for multiple testing.

The analysis and visualization were performed using GraphPad Prism v10.2.0 (GraphPad Software, San Diego, CA, USA). For data obtained with RT-qPCR, the comparison of miRNA Ct values between two groups was performed using a Mann-Whitney U test. ns = not significant, significant differences between groups are denoted by * *p* < 0.05, ** *p* < 0.01, *** *p* < 0.001, or **** *p* < 0.0001. ROC curve analyses were performed in GraphPad Prism v10.2.0 using normalized counts or Ct values as input. The area under the curve (AUC) was calculated to assess the diagnostic performance of individual miRNAs and their ratios. AUCs and 95% confidence intervals were calculated using the nonparametric ROC method implemented in GraphPad Prism.

## Results

**Fig. 1:**
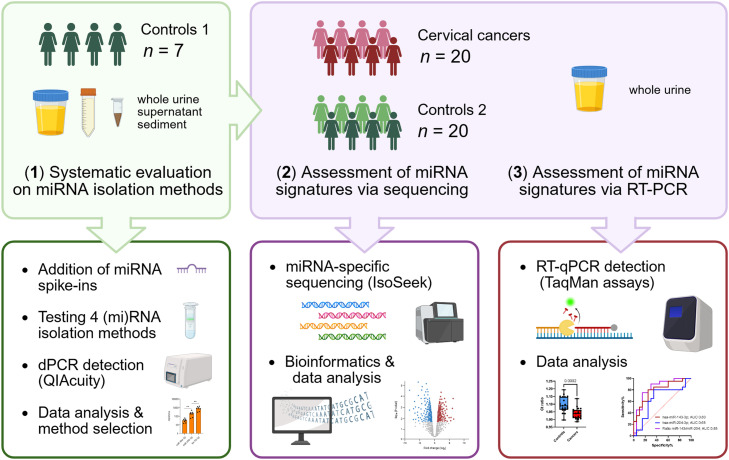
**Study design and workflow. (**1) A first series of seven urine samples from healthy controls was used for a systematic evaluation of miRNA isolation methods for different urine fractions (whole urine, supernatant, and sediment). (**2**) A second series of whole urine samples from twenty controls and twenty cancer patients was assessed using small RNA sequencing (IsoSeek). (**3**) Candidate miRNA signatures identified through IsoSeek were subsequently assessed using the same samples from phase 2 via RT-qPCR. This figure was made using BioRender (www.biorender.com).

To evaluate the feasibility of urinary miRNA profiling and their biomarker potential for cervical cancer detection, a three-step approach was undertaken, as outlined in Fig. 1. The study consisted of: 1) a systematic evaluation of four miRNA isolation methods applied to different urine fractions (whole urine, supernatant, and sediment) using samples collected from healthy control series 1 (*n* = 7) and dPCR; 2) the assessment of urinary miRNAs via small RNA sequencing (IsoSeek) performed on whole urine isolates of urine samples collected from cancer patients (*n* = 20, **Supplementary Table 3**) and age-matched healthy control series 2 (*n* = 20), isolated using Norgen RNA kit; and 3) analytical confirmation of urinary miRNA signatures via RT-qPCR (*n* *=* *40*) using the same isolates from 2), except for five controls from which insufficient RNA was remaining and were replaced by five alternative controls.

### Performance of miRNA isolation methods differs by urine fractions

To select the most suitable miRNA isolation method for each urine fraction, we evaluated four isolation methods referred to as miRNeasy_m, miRNeasy_sp, Norgen, and TRIzol using urine samples collected from healthy controls (*n* = 7) (Fig. 1). These samples were processed to obtain three fractions: whole urine, supernatant, and sediment. The performance of the isolation methods was assessed on four aspects: total miRNA yield, PCR inhibition, spike-ins yield, and the yield of certain endogenous miRNAs.

**Fig. 2:**
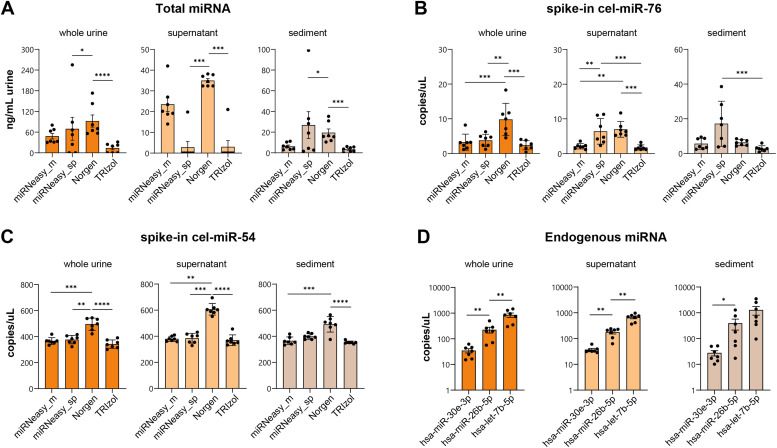
**Systematic analysis of miRNA isolation methods in urine.** Comparison analyses of four miRNA isolation methods in whole urine, supernatant, and sediment fractions in a series of seven healthy controls via dPCR. **(A)** The analyses involve assessing the total miRNA yield. **(B)** The yield of the spike-in cel-miR-76–3p added during miRNA isolation. **(C)** The yield of the spike-in cel-miR-54–3p added during reverse transcription (RT). **(D)** and the measurement of low (hsa-miR-30e-3p), medium (hsa-miR-26b-5p), and high (hsa-let-7b-5p) abundant endogenous miRNAs. Paired differences in (**A), (B),** and **(C)** were analyzed with A Friedman test, and unpaired differences in (**D)** were analyzed with a Kruskal-Wallis test, followed by the Benjamini-Hochberg test correction. A *q* < 0.10 is considered significant and displayed in figures. * *q* < 0.10, ** *q* < 0.05, *** *q* < 0.01, or **** *q* < 0.001.

The miRNA yield was calculated as the amount of miRNA (ng) per mL of urine input. The yield showed considerable variation across all three urine fractions and all four isolation methods (Fig. 2**A**). For whole urine and supernatant, higher miRNA yields were obtained using Norgen, which performed better compared to TRIzol and miRNeasy_sp (*q* < 0.10). The miRNeasy_sp performed better than TRIzol for the sediment fraction (*q* < 0.10). In terms of total RNA, miRNeasy_m (*q* < 0.10, Friedman test) yielded significantly more total RNA from whole urine and supernatant, whereas the highest yields were obtained with miRNeasy_sp (*q* < 0.05) in urine sediment fractions (**Supplementary Fig. 1A**). By calculating the miRNA/RNA ratio, we found that Norgen provided a higher relative fraction of miRNAs from total RNA in both whole urine (*q* < 0.10) and supernatant (*q* < 0.10) compared to the other isolation methods (*q* < 0.10). For the urine sediment, Norgen performed as well as miRNeasy_sp (**Supplementary Fig. 1B**).

To further evaluate the performance of miRNA isolation, the spike-in control cel-miR-76–3p (added before RNA isolation) was quantified via dPCR. Norgen isolated significantly more copies of cel-miR-76–3p in the whole urine fraction compared to other isolation methods (*q* < 0.05) (Fig. 2**B**). Similarly, in the supernatant fraction, Norgen isolated significantly more copies of cel-miR-76–3p compared to miRNeasy_m and TRIzol (*q* < 0.05) but showed comparable performance to miRNeasy_sp (Fig. 2**B**). For the sediment fraction, the miRNeasy_sp method isolated more copies of cel-miR-76–3p than TRIzol (*q* < 0.01). Next, to assess PCR inhibition, RT spike-in cel-miR-54–3p (added during reverse transcription) was measured via dPCR. In general, Norgen-derived isolates exhibited significantly more cel-miR-54–3p copies than all the other isolates for all three urine fractions (*q* < 0.05) (Fig. 2**C**), representing the lowest PCR inhibition, but the difference between Norgen and miRNeasy_sp for the sediment was not significant (*q* = 0.2356).

Finally, we quantified endogenous miRNAs of varying abundance using dPCR on urine fractions isolated with either Norgen (whole urine/supernatant) or miRNeasy_sp (sediment). Based on preliminary in-house data, we selected the miRNAs hsa-miR-30e-3p, hsa-miR-26b-5p, and hsa-let-7b-5p as potentially low, medium, and high abundant miRNAs in urine, respectively. As expected, we observed that the relative abundance of hsa-miR-30e-3p, hsa-miR-26b-5p, and hsa-let-7b-5p was low, medium, and high, respectively, in whole urine and supernatant fractions (*q* < 0.05, Kruskal-Wallis test) (Fig. 2**D**).

### Sequencing and PCR-based assessment of urinary miRNA signatures for cervical cancer

IsoSeek is a sequencing tool for miRNA profiling that reduces ligation and PCR amplification bias and improves the accuracy of miRNA detection in low-input samples, such as urine [14]. To assess the potential of urinary miRNA biomarker discovery by IsoSeek, we analyzed Norgen-derived isolates of whole urine samples from twenty healthy controls and twenty cervical cancer patients.

**Fig. 3:**
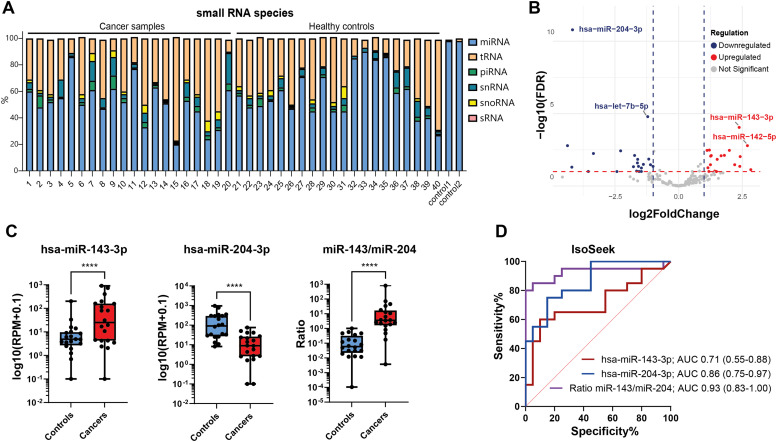
**Urinary small RNA profiles and identification of cancer-associated miRNA biomarkers. (A)** Distribution of small RNA classes in whole urine from healthy controls (*n* = 20) and cervical cancer patients (*n* = 20) that were sequenced via IsoSeek. **(B)** Volcano plot showing differentially expressed urinary miRNAs in cervical cancers versus healthy controls. Upregulated miRNAs are shown in red, downregulated miRNAs are shown in blue. A log2FoldChange = ±1 was set as a threshold and displayed as a blue vertical dashed line, and an FDR < 0.1 was set as a significant threshold and displayed as a red horizontal dashed line. Key deregulated miRNAs are annotated. **(C)** Boxplots showing normalized counts of hsa-miR-143–3p, hsa-miR-204–3p, and hsa-miR-143–3p/hsa-miR-204–3p ratio in healthy controls (*n* = 20) and cancers (*n* = 20) measured by IsoSeek. For hsa-miR-143–3p and hsa-miR-204–3p, the statistical comparison of differential expression was displayed; an FDR < 0.10 is considered significant and displayed in figures. **** FDR <0.0001. For the ratio of hsa-miR-143–3p/hsa-mir-204–3p, a p-value was calculated by an unpaired Mann-Whitney test, **** *p* < 0.0001. **(D)** ROC curves for sequencing-derived normalized counts for hsa-miR-143–3p (red), hsa-miR-204–3p (blue), and the hsa-miR-143–3p/hsa-miR-204–3p ratio (purple). 95% confidence intervals are indicated in brackets. AUC: area under the curve; FDR: false discovery rate; RPM: reads per million.

The composition of RNA species showed considerable variation between individual samples, with miRNA proportions ranging from approximately 20% to 90% of total small RNAs (Fig. 3**A**). Despite this variability in small RNA composition, we consistently detected >190 distinct mature miRNAs (range 117–537) in each sample (**Supplementary Table 4**).

After filtration, 237 distinct mature miRNAs were included in differential expression analysis, which revealed distinct miRNA signatures associated with cervical cancer (Fig. 3**B**). Nineteen miRNAs were significantly upregulated in the urine of cervical cancer patients, including hsa-miR-143–3p (FDR <0.0001) and hsa-miR-142–5p (FDR <0.01). Twenty-one miRNAs were downregulated in cancer urine, including hsa-miR-204–3p (FDR < 0.0001) and hsa-let-7b-5p (FDR < 0.0001 (Fig. 3**C**, Table 1).

**Table 1:** Differential expression of sequencing-based urinary miRNA in cervical cancer patients compared to healthy controls. Assessed miRNAs are indicated in bold.

**Upregulated**				
**miRNA**	**log2FoldChange**	**lfcSE**	**pvalue**	**FDR**
**hsa-miR–143–3p**	2.378605685	0.494594	1.52E-06	8.94E-05
hsa-miR–142–5p	2.701330227	0.662975	4.61E-05	0.001632
hsa-miR-103a-3p	1.208879261	0.312812	0.000111	0.003283
hsa-miR–223–3p	1.993176251	0.526888	0.000155	0.003431
hsa-miR–25–3p	1.117749658	0.295481	0.000155	0.003431
hsa-miR-146a-5p	1.797558672	0.517589	0.000515	0.007593
hsa-miR–191–5p	1.282949616	0.374738	0.000618	0.007813
hsa-miR–140–3p	1.66896515	0.491597	0.000686	0.008099
hsa-miR-92a-3p	1.258145816	0.374424	0.000779	0.008616
hsa-miR–340–5p	2.418283594	0.727619	0.000889	0.009254
hsa-miR-106b-3p	1.515638512	0.480782	0.001619	0.014368
hsa-miR-6126	2.20273897	0.784131	0.004967	0.033816
hsa-miR–24–3p	1.250044571	0.442914	0.004768	0.033816
hsa-miR-4492	2.402794721	0.894475	0.007226	0.043848
hsa-miR-146b-5p	1.151744124	0.448147	0.010169	0.054545
hsa-miR-3648	2.834934941	1.160823	0.014599	0.073829
hsa-miR-20a-5p	1.419360315	0.612069	0.020397	0.092573
hsa-miR-1246	1.190168756	0.528457	0.024312	0.098612
hsa-miR-199a-3p	1.487305128	0.667404	0.025848	0.099631

**Downregulated**				

**miRNA**	**log2FoldChange**	**lfcSE**	**pvalue**	**FDR**

**hsa-miR–204–3p**	−4.167355428	0.559247	9.21E-14	1.63E-11
hsa-let-7b-5p	−1.212925658	0.232537	1.83E-07	1.62E-05
hsa-miR–4800–5p	−4.36442521	1.068177	4.39E-05	0.001632
hsa-miR-184	−2.276918093	0.609786	0.000188	0.003707
hsa-miR-4484	−3.34557174	0.935934	0.000351	0.005645
hsa-miR-320a-3p	−1.72925305	0.50162	0.000566	0.007708
hsa-miR–192–5p	−1.172496567	0.367141	0.001405	0.013817
hsa-miR–423–5p	−1.607649373	0.510098	0.001624	0.014368
hsa-miR-320b	−1.618006658	0.545275	0.003004	0.02532
hsa-miR-378a-3p	−1.382815933	0.482364	0.004147	0.031915
hsa-miR-99a-5p	−1.104527444	0.391992	0.004836	0.033816
hsa-miR-193b-5p	−1.575838075	0.588692	0.007432	0.043848
hsa-let-7e-5p	−1.005150186	0.370658	0.006692	0.043848
hsa-miR-4443	−1.774064889	0.669912	0.008092	0.046203
hsa-miR-4739	−4.193429469	1.604609	0.008966	0.049591
hsa-miR–574–5p	−1.460883845	0.573615	0.010871	0.056595
hsa-miR-378f	−1.649657945	0.710015	0.020157	0.092573
hsa-miR–483–5p	−3.546942611	1.565813	0.023498	0.098612
hsa-miR-378 *g*	−1.552057006	0.685991	0.023666	0.098612
hsa-miR-378i	−1.463712921	0.64716	0.023713	0.098612
hsa-miR-3182	−2.423030916	1.087626	0.025893	0.099631

We further examined the biomarker potential of the most significant up- and downregulated miRNAs, hsa-miR-143–3p and hsa-miR-204–3p. Exploratory receiver operating characteristic (ROC) analysis demonstrated that both miRNAs have biomarker potential (Fig. 3**D**). hsa-miR-204–3p achieved an AUC of 0.86 (95% CI 0.75–0.97), while hsa-miR-143–3p reached an AUC of 0.71 (95% CI 0.55–0.88). Importantly, combining the two markers as a ratio (hsa-miR-143–3p/hsa-miR-204–3p) further improved discrimination between cancer and controls, yielding an AUC of 0.93 (95% CI 0.83–1.00).

**Fig. 4:**
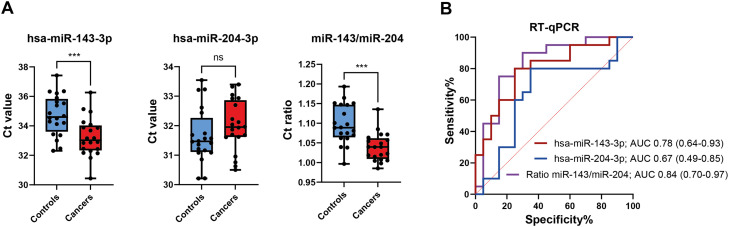
**Analysis of urinary miRNA profiles by RT-qPCR. (A)** Boxplots showing Ct value of hsa-miR-143–3p, hsa-miR-204–3p, and hsa-miR-143–3p/hsa-miR-204–3p ratio in healthy controls (*n* = 20) and cancers (*n* = 20) measured by RT-qPCR. Unpaired comparisons were analyzed with a Mann-Whitney test, ns: non-significant, *** *p* < 0.001. **(B)** ROC curves for RT-qPCR Ct values for hsa-miR-143–3p (red), hsa-miR-204–3p (blue), and the hsa-miR-143–3p/hsa-miR-204–3p ratio (purple). 95% confidence intervals are indicated in brackets. AUC: area under the curve.

RT-qPCR assessment on the same samples confirmed these findings: hsa-miR-143–3p was significantly upregulated (with lower Ct values) in cancers compared to controls, and a non-significant downregulation of hsa-miR-204–3p (with higher Ct values) was seen in cancers (Fig. 4**A**). This is consistent with the correlation analysis between RT-qPCR Ct values and IsoSeek-derived RPM values; hsa-miR-143–3p demonstrate a significant correlation, whereas hsa-miR-204–3p is not significant (**Supplementary Fig. 2**). Combined, the Ct ratio of hsa-miR-143/hsa-miR-204 was significantly lower in cancer samples (*p* < 0.001; Fig. 4**A**), and ROC analysis achieved an AUC of 0.84 (95% CI 0.70–0.97), higher than either miRNA alone (Fig. 4**B**). Together, these results highlight the complementary value of hsa-miR-143–3p and hsa-miR-204–3p, with their ratio providing the strongest discriminatory potential for distinguishing cancer patients from healthy controls.

## Discussion

Our systematic evaluation of miRNA isolation methods demonstrated that their performance varied significantly across different urine fractions (whole urine, supernatant, and sediment). These findings underscore the necessity of selecting appropriate isolation methods tailored to specific urine fractions to maximize miRNA recovery and minimize inhibition for downstream applications [25]. This optimization is crucial for ensuring the accuracy and reliability of subsequent miRNA analyses. For instance, the Norgen method's low PCR inhibition and high miRNA yield in whole urine and supernatant fractions make it a robust choice for these sample types, which is consistent with previous reports [11,26]. Similarly, the efficacy of miRNeasy_sp in isolating urine sediment highlights its suitability for capturing miRNAs from this fraction. Our findings, therefore, provide a methodological benchmark for urinary miRNA research and emphasize the importance of optimized, fraction-specific protocols to enhance data quality and reproducibility.

The application of IsoSeek, an advanced miRNA-specific sequencing approach [14,16], enabled profiling of urinary miRNAs from women with cervical cancer and healthy controls. To our knowledge, this represents the first application of sequencing-based miRNA profiling and marker discovery in urine samples in the context of cervical cancer [10]. These findings highlight the suitability of IsoSeek for miRNA detection and quantification in low-input samples, as previously reported [14,16]. The detectability of miRNAs in urine is consistent with findings from other groups [27,28]. For instance, the recent study of Calfa et al. [27] employed a targeted nanoString panel assessing pre-selected miRNAs. Nonetheless, our use of advanced RNA sequencing provides a discovery-driven approach that is particularly advantageous for comprehensively characterizing the miRNA in low-abundance samples such as urine, without prior assumptions about relevant biomarkers. Given the low abundance of miRNAs in urine, we applied relatively permissive filtering criteria, including miRNAs with ≥10 counts in at least two of the 40 samples analyzed. Our analyses identified several differentially expressed miRNAs, with hsa-miR-143–3p significantly upregulated and hsa-miR-204–3p significantly downregulated in urine from cervical cancer patients compared to healthy controls. Both miRNAs have previously been implicated in cancer biology: hsa-miR-143–3p has been associated with oncogenic processes, including cell proliferation and invasion, with context-dependent functions across different cancer types [29,30]. However, while downregulation of hsa-miR-143–3p has been reported in cervical cancer tissue [31], its elevation in patient urine may reflect selective secretion by cancer-associated cells or circulating tumor-derived exosomes, highlighting the distinct biology of urinary versus tissue compartments. Conversely, hsa-miR-204–3p has been characterized as a tumor suppressor whose downregulation correlates with poor prognosis in multiple cancers [32]. Importantly, combining these two biomarkers as a ratio (miR-143/miR-204) yielded superior discriminatory power compared to either marker alone, highlighting the complementary value of their reciprocal expression patterns. This ratio-based approach was confirmed by RT-qPCR and may provide a more robust biomarker framework, as ratio metrics can normalize technical variation and amplify biological differences [33].

Notably, we observed a large inter-sample variation in miRNA concentrations, which likely reflects both variation in the miRNA expression in cervical cells and differences in the amount of cells shed into urine [34,35]. A variation in miRNA signatures related to the amount of cervical cells in different sample types was also observed in our previous studies on self-collected cervicovaginal swabs or cervical scrapes [7,8]. These sample-type-dependent miRNA signatures further support the use of a sequencing-based strategy for the discovery of urinary miRNA signatures. Similarly, the urinary miRNA biomarkers identified in our study differ from those reported by others [10], which might be explained by our unprecedented sequencing discovery approach. However, our small sample size precludes definitive conclusions, and these observations warrant validation in larger, independent cohorts.

Our results highlight the potential of urinary miRNAs as non-invasive biomarkers for cervical cancer, with significant implications for improving cancer screening. The ease of home-based urine collection [25], combined with the stability of miRNAs in urine [36], makes this approach particularly attractive for widespread clinical use, especially in low- and middle-income countries where traditional screening and triage methods may be less accessible [37,38]. Additionally, integrating urinary miRNA profiling with existing screening and triage strategies, including HPV DNA testing [38,39], HPV genotyping [40,41], or other markers such as DNA methylation [4,5], could enhance early detection rates and improve patient outcomes. However, as emphasized in recent standardization efforts for urine-based liquid biopsies, including the MUMIE reporting framework [42], which informed the design and reporting of our study, translation to the clinical practice will require: (1) standardized pre-analytical protocols for urine collection, processing, and storage; (2) consensus guidelines for miRNA isolation and quantification methods; (3) comprehensive reporting of technical parameters including urine fraction analyzed, input volume, and isolation method; and (4) validation in large, prospective studies with well-defined clinical endpoints. Addressing these requirements will be critical for ensuring reproducibility across laboratories and enabling meaningful cross-study comparisons [25].

The strengths of this study include a comprehensive head-to-head comparison of miRNA isolation methods across urine fractions using dPCR read-outs, combined with various analytical approaches (small RNA sequencing and RT-qPCR) to investigate the potential of urinary biomarkers. Several limitations of our study should also be considered. First, the sample size was relatively small, consisting of 20 cervical cancer cases and 20 controls. Second, biomarker discovery and RT-qPCR confirmation were performed in the same cohort, which may lead to optimistic estimates of diagnostic performance. Therefore, the reported ROC performance estimates should be considered exploratory until validated in independent cohorts. Third, clinically relevant intermediate groups, such as hrHPV-positive women without cancer or women with cervical precancer, were not included. Fourth, urinary miRNA profiles may be influenced by multiple biological and pre-analytical variables, including urine concentration, cellularity, collection timing, menstruation, inflammation, renal function, and the degree of cervicovaginal cell shedding into urine. Finally, standardized protocols for urine processing, normalization, and quality control will be required before translation into routine clinical practice [25,42]. As such, further research with a larger sample size, including urine samples of women with precancer lesions, is needed to further investigate the clinical utility of the present findings.

In conclusion, this technical optimization and feasibility study establishes the foundation for developing urinary miRNAs as non-invasive biomarkers for cervical cancer and potentially other cancers of the urogenital tract. By demonstrating the possibility of sequencing-based biomarker discovery in urine and identifying promising candidate markers, our work contributes to the growing body of evidence supporting the use of urine-based liquid biopsies.

## Ethical approvals

Ethical approval was provided by the Medical Ethical Committee of the VU University Medical Center (URIC biobank, reference number: 2018.657; and SOLUTION studies, reference number 2016.213; Trial registration ID: NL56664.029.16).

## Availability of data

The datasets generated and/or analyzed during the current study are available from the corresponding author on reasonable request.

## Funding

This research was supported by the China Scholarship Council Grant Number 202008420215 and the Stichting NEXTGEN HIGHTECH Program (Biomed02).

## CRediT authorship contribution statement

**Mengfei Xu:** Conceptualization. **Mariano A. Molina:** Conceptualization. **Cristina Gómez-Martín:** Formal analysis. **Maaike M.H.S. Dekker:** Methodology. **Saba Ghasemi:** Formal analysis. **Johan de Rooij:** Resources. **D. Michiel Pegtel:** Methodology. **Nienke E. van Trommel:** Resources. **Renske D.M. Steenbergen:** Supervision, Resources, Conceptualization.

## Declaration of competing interest

Renske D. M. Steenbergen has a minority stake in Self-screen B.V., a spin-off company of Amsterdam UMC, location VUmc, Amsterdam, The Netherlands. Michiel Pegtel serves as a paid advisor to Y2Y BV, a urinary diagnostics company. The remaining authors declare no conflict of interest.

## Appendix Supplementary materials

**Supplementary Table 1.** Summary of sequencing data, preprocessing and miRNA mapping metrics. Image, application 1

Image, application 2

**Supplementary Fig. 1. Total RNA and miRNA/RNA ratios.** Additional comparison analyses of four miRNA isolation methods in whole urine, supernatant, and sediment fractions in healthy control series 1 via dPCR. The analyses involve assessing **(A)** the total RNA yield and **(B)** the fraction of miRNAs from the total RNA (miRNA/RNA ratio). Paired differences were analyzed with a Friedman test, followed by the Benjamin-Hochberg test correction. A *q* < 0.10 is considered significant. * *q* < 0.10, ** *q* < 0.05, *** *q* < 0.01, or **** *q* < 0.001. Image, image 3 dummy alt text

**Supplementary Fig. 2**. Correlation between RT-qPCR CT values and small RNA sequencing RPM for hsa-miR-143–3p and hsa-miR-204–3p Scatter plots showing the correlation between CT values obtained by RT-qPCR and read counts per million (RPM) obtained by small RNA sequencing for hsa-miR-143–3p (left) and hsa-miR-204–3p (right). RPM values were transformed as log₂(RPM + 0.1) to accommodate zero values. Each dot represents one urine sample, coloured by group (red: cervical cancer *n**=**20*; blue: control *n**=**15*). The dashed line indicates the linear regression fit. Pearson correlation coefficient (r) and corresponding p-value are shown. Image, image 4 dummy alt text
