# Supplementary material for: Discovery and analytical assessment of urinary miRNA biomarkers for cervical cancer using advanced small RNA sequencing
Source: Transl Oncol. 2026 Jun 16;71:102857. doi: 10.1016/j.tranon.2026.102857 (PMC13292658; doi:10.1016/j.tranon.2026.102857)
Supplement: Supplementary file 2 [file mmc2.docx]

**Supplementary materials**

**Supplementary Table 2.** TaqMan™ MicroRNA Assay

| miRNA | Assay ID |
| --- | --- |
| cel-miR-54-5p | 001361 |
| cel-miR-76-3p | 000229 |
| hsa-let-7b-5p | 002619 |
| hsa-miR-15b-5p | 000390 |
| hsa-miR-26b-5p | 000406 |
| hsa-miR-30e-3p | 000422 |
| hsa-miR-143-3p | 002249 |
| hsa-miR-204-3p | 463101_mat |

**Supplementary Table 3.** Clinical characteristics of the SOLUTION-1 cohort.

| Age (years) |  |
| --- | --- |
| Median (range) | 49 (30-75) |
|  |  |
| Histological Type |  |
| Squamous cell carcinoma | 16 (80%) |
| Adenocarcinoma | 3 (15%) |
| Adenosquamous carcinoma | 1 (5%) |
|  |  |
| FIGO Stage (2018) |  |
| *Stage I* | *11 (55%)* |
| IB1 | 4 (20%) |
| IB2 | 2 (10%) |
| IB3 | 5 (25%) |
| *Stage II* | *5 (25%)* |
| IIA1 | 1 (5%) |
| IIA2 | 1 (5%) |
| IIB | 3 (15%) |
| *Stage III* | *3 (15%)* |
| IIIB | 2 (10%) |
| IIIC1 | 1 (5%) |
| *Stage IV* | *1 (5%)* |

**Supplementary Table 4.** Summary of miRNA detection in each urine sample.

| **Sample** | **Group** | **Detected_miRNAs** | **Mean_RPM** | **Max_RPM** |
| --- | --- | --- | --- | --- |
| M01 | control | 256 | 2.99 | 1040.93 |
| M02 | control | 255 | 3.26 | 1588.09 |
| M03 | control | 350 | 0.65 | 223.43 |
| M04 | control | 230 | 3.98 | 1384.41 |
| M05 | control | 354 | 2.46 | 1145.47 |
| M06 | control | 266 | 4.69 | 1436.82 |
| M07 | control | 292 | 10.74 | 4716.96 |
| M08 | control | 317 | 1.16 | 287.57 |
| M09 | control | 281 | 11.99 | 3711.65 |
| M10 | control | 242 | 4.01 | 2056.01 |
| M11 | control | 297 | 7.6 | 2813.19 |
| M12 | control | 537 | 50.63 | 21626.22 |
| M13 | control | 324 | 44.17 | 16404.49 |
| M14 | control | 382 | 20.67 | 8283.5 |
| M15 | control | 340 | 12.24 | 5694.98 |
| MA_01 | control | 167 | 4.78 | 2204.66 |
| MA_02 | control | 258 | 4.77 | 1735.69 |
| MA_03 | control | 173 | 0.76 | 268.29 |
| MA_04 | control | 276 | 1.99 | 456.18 |
| MA_05 | control | 176 | 1.26 | 504.8 |
| M16 | cancer | 193 | 2.35 | 796.13 |
| M17 | cancer | 153 | 1.91 | 1342.46 |
| M18 | cancer | 216 | 1.43 | 308.98 |
| M19 | cancer | 276 | 9.72 | 5309.71 |
| M20 | cancer | 306 | 42.78 | 14547.53 |
| M21 | cancer | 117 | 1.07 | 205.47 |
| M22 | cancer | 450 | 33.1 | 31097.71 |
| M23 | cancer | 297 | 22.22 | 5433.71 |
| M24 | cancer | 459 | 47.4 | 31600.49 |
| M25 | cancer | 223 | 1.56 | 639.64 |
| M26 | cancer | 274 | 26.43 | 12243.05 |
| M27 | cancer | 298 | 11.84 | 1437.08 |
| M28 | cancer | 313 | 13.04 | 4237.22 |
| M29 | cancer | 168 | 10.92 | 3916.06 |
| M30 | cancer | 362 | 3.2 | 914.93 |
| MA_06 | cancer | 216 | 1.57 | 1731.17 |
| MA_07 | cancer | 247 | 1.07 | 444.56 |
| MA_08 | cancer | 258 | 3.88 | 689.85 |
| MA_09 | cancer | 252 | 0.48 | 124.07 |
| MA_10 | cancer | 283 | 10.85 | 14672.52 |
