# Supplementary figures and images for: Discovery and analytical assessment of urinary miRNA biomarkers for cervical cancer using advanced small RNA sequencing
Source: Transl Oncol. 2026 Jun 16;71:102857. doi: 10.1016/j.tranon.2026.102857 (PMC13292658; doi:10.1016/j.tranon.2026.102857)

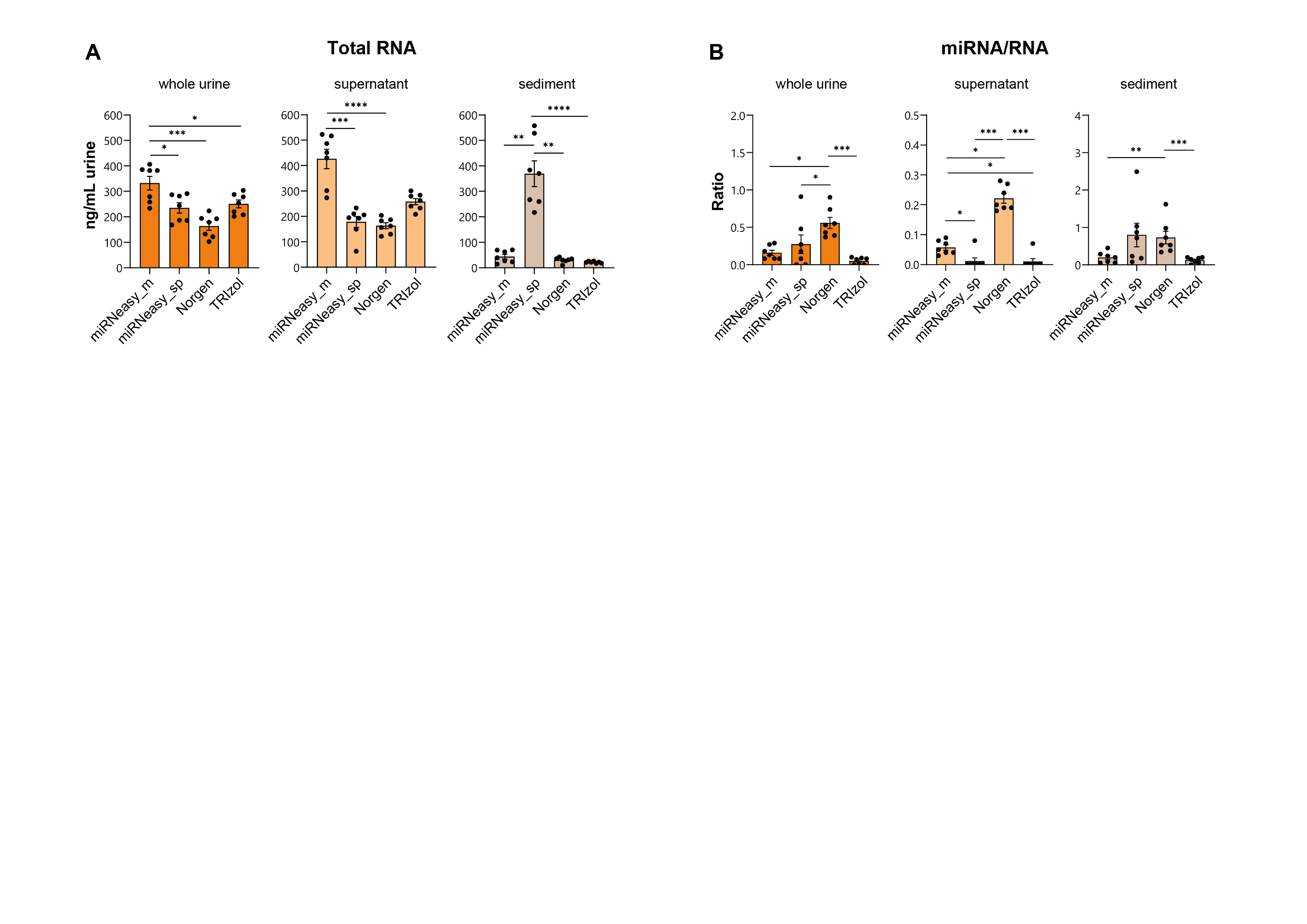

Supplement: Supplementary file 3 — Supplementary Fig. 1. Total RNA and miRNA/RNA ratios. Additional comparison analyses of four miRNA isolation methods in whole urine, supernatant, and sediment fractions in healthy control series 1 via dPCR. The analyses involve assessing (A) the total RNA yield and (B) the fraction of miRNAs from the total RNA (miRNA/RNA ratio). Paired differences were analyzed with a Friedman test, followed by the Benjamin-Hochberg test correction. A q < 0.10 is considered significant. * q < 0.10, ** q < 0.05, *** q < 0.01, or **** q < 0.001. [file mmc3.jpg]

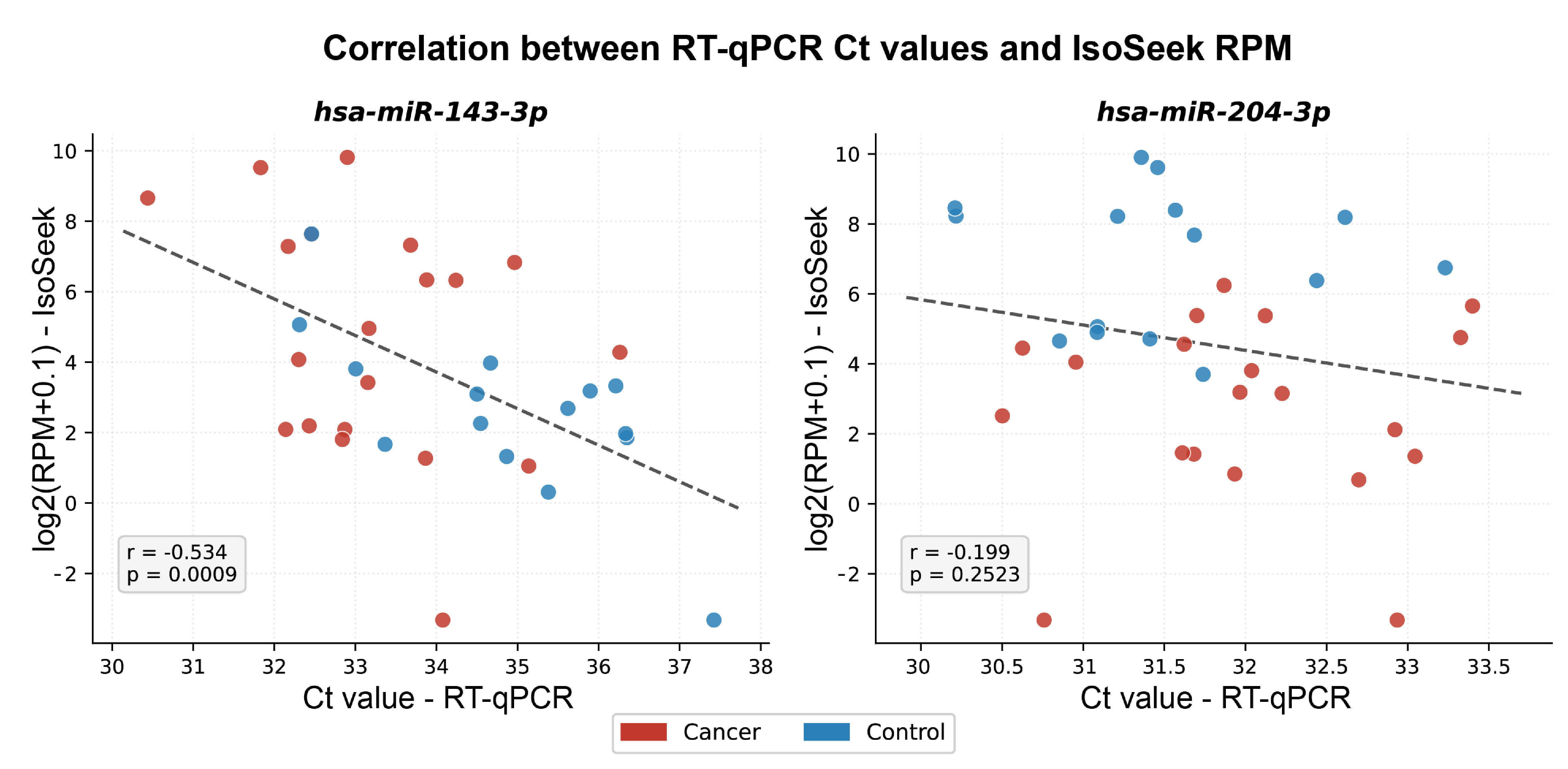

Supplement: Supplementary file 4 — Supplementary Fig. 2. Correlation between RT-qPCR CT values and small RNA sequencing RPM for hsa-miR-143–3p and hsa-miR-204–3p Scatter plots showing the correlation between CT values obtained by RT-qPCR and read counts per million (RPM) obtained by small RNA sequencing for hsa-miR-143–3p (left) and hsa-miR-204–3p (right). RPM values were transformed as log₂(RPM + 0.1) to accommodate zero values. Each dot represents one urine sample, coloured by group (red: cervical cancer n=20; blue: control n=15). The dashed line indicates the linear regression fit. Pearson correlation coefficient (r) and corresponding p-value are shown. [file mmc4.jpg]
